# Crystal structure of 4-(3,4-di­cyano­phen­oxy)-*N*-[3-(di­methyl­amino)­prop­yl]benzamide mono­hydrate: a phen­oxy­phthalo­nitrile derivative

**DOI:** 10.1107/S2056989015014991

**Published:** 2015-08-15

**Authors:** Senem Çolak, Salih Zeki Yıldız, Nagihan Çaylak Delibaş, Hasan Pişkin, Tuncer Hökelek

**Affiliations:** a Department of Chemistry, Sakarya University, 54187 Esentepe, Sakarya, Turkey; b Department of Physics, Sakarya University, 54187 Esentepe, Sakarya, Turkey; c Department of Physics, Hacettepe University, 06800 Beytepe, Ankara, Turkey

**Keywords:** crystal structure, amido amine derivatives, phthalo­nitrile derivatives, hydrogen bonding

## Abstract

In the title compound, C_20_H_20_N_4_O_2_·H_2_O, the planes of the phen­oxy and phthalo­nitrile rings are oriented at a dihedral angle of 60.39 (5)°. The 3-(di­­methyl­amino)­propyl chain has an extended conformation and is *cis* with respect to the phthalo­nitrile ring. In the crystal, O—H⋯O, O—H⋯N and N—H⋯O hydrogen bonds link the mol­ecules to form slabs parallel to (100). There are also C—H⋯O and C—H⋯N hydrogen bonds and C—H⋯π inter­actions present within the slabs. The slabs are linked by a pair of inversion-related C—H⋯N hydrogen bonds, involving phthalo­nitrile rings, forming a three-dimensional structure.

## Abstract 2

In the title compound, the planes of the phen­oxy and phthalo­nitrile rings are oriented at a dihedral angle of 60.39 (5)°. In the crystal, mol­ecules are linked by O—H⋯O, O—H⋯N and N—H⋯O hydrogen bonds, forming slabs parallel to (100). The slabs are linked by a pair of inversion-related C—H⋯N hydrogen bonds, forming a three-dimensional structure.

## Chemical context   

Amido amine derivatives are suggested as exhibiting an outstanding combination of surfactant properties. Well-known application fields for amino derivatives are their use as synthetic inter­mediates of anti­cancer agents, anti­biotics and other drugs. They also exhibit exceptionally low ocular irritation and oral toxicity, being well tolerated by human tissue (Roy *et al.*, 2010 ▸). Amides and amido amines of fatty acids and polyamine products are used as typical corrosion inhibitors in high dosage, despite their poor biodegradability, because of their extremely good oil solubility. Polyamines play an important role in cell growth and bind to the phosphate residues of DNA, stabilizing the specific conformation of the latter (Karaoğlan *et al.*, 2011 ▸; Göksel *et al.*, 2013 ▸; Kim *et al.*, 2012 ▸; Çolak *et al.*, 2014 ▸). In this context, we synthesized 4-(3,4-di­cyano­phen­oxy)-*N*-[3-(di­methyl­amino)­prop­yl]benzamide monohydrate and report herein on its crystal structure.

## Structural commentary   

**Figure 1:**
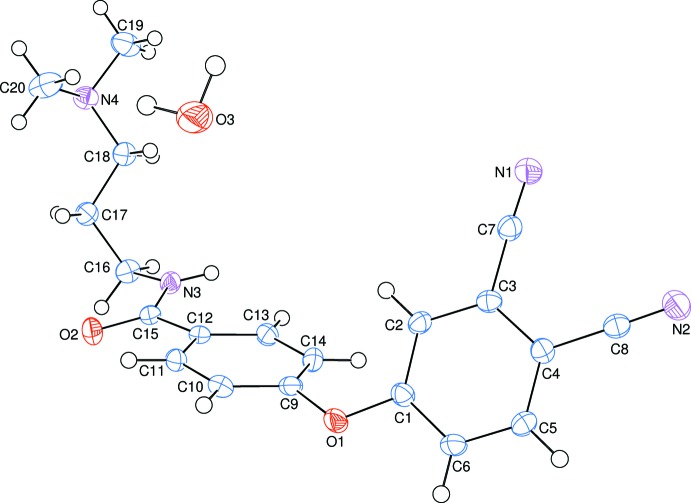
The mol­ecular structure of the title compound, showing the atom labelling. Displacement ellipsoids are drawn at the 50% probability level.

The mol­ecular structure of the title compound, which crystallized as a monohydrate, is illustrated in Fig. 1 ▸. The phthalo­nitrile (A = atoms C1–C6) and phen­oxy (B = atoms C9–C14) rings are oriented at a dihedral angle of 60.39 (5)°. Atoms O1 N1, N2, C7 and C8 are at distances of 0.0799 (13), −0.1207 (18), 0.0366 (18), −0.0613 (19) and 0.0183 (18) Å, respectively, from phthalo­nitrile ring A, and are thus almost coplanar with this ring. In contrast, atoms O1, N3 and C15 are displaced by −0.1329 (13), 0.1004 (15) and −0.1247 (17) Å, respectively, from phen­oxy ring B. The mean plane of the amide group (C15/O2/N3) makes a dihedral angle of 15.8 (2)° with that of phen­oxy ring B. The 3-(di­methyl­amino)­propyl chain [N4/C16–C18; maximum deviation = 0.057 (2) Å] has an extended conformation and its mean plane is inclined to ring B by 68.53 (16)°, and by 28.69 (16)° to phthalo­nitrile ring A.
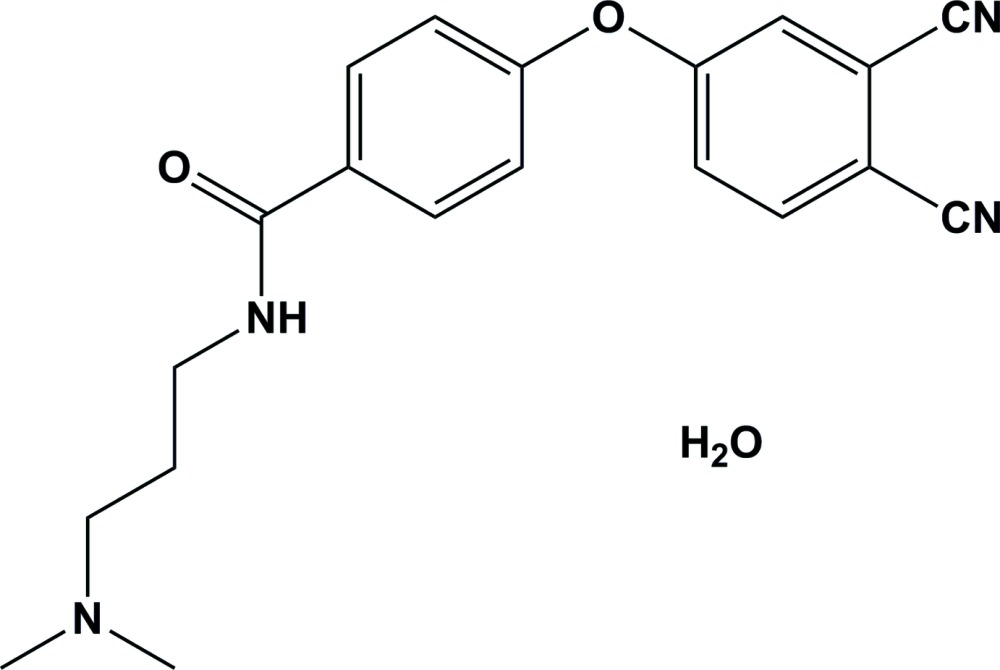


## Supra­molecular features   

**Figure 2:**
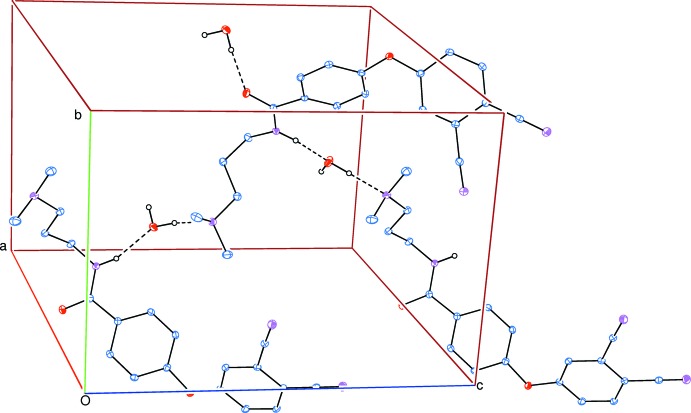
Part of the crystal packing of the title compound. The O—H⋯O, O—H⋯N and N—H⋯O hydrogen bonds are shown as dashed lines (see Table 1 ▸). Only H atoms involved in hydrogen bonding have been included for clarity.

**Figure 3:**
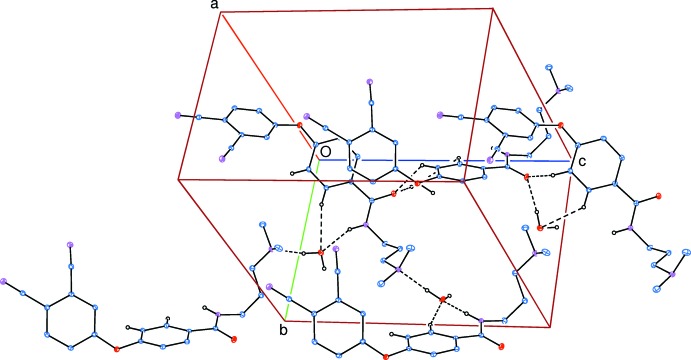
A partial view of the crystal packing of the title compound. The N—H_amd_⋯O_w_, O—H_w_⋯O_amd_, O—H_w_⋯N_dma_, C—H_phen_⋯O_amd_ and C—H_phen_⋯O_w_ (amd = amide, dma = di­methyl­amino, w = water and phen = phen­oxy) hydrogen bonds, enclosing 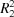
(7) and 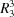
(7) ring motifs, are shown as dashed lines (see Table 1 ▸). Only H atoms involved in hydrogen bonding have been included for clarity.

**Figure 4:**
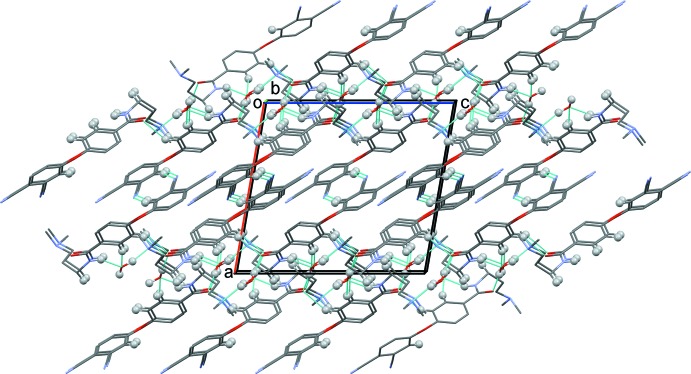
A view along the *b* axis of the crystal packing of the title compound. The hydrogen bonds are shown as dashed lines (see Table 1 ▸). Only H atoms involved in hydrogen bonding (grey balls) have been included for clarity.

In the crystal, N—H_amd_⋯O_w_ (amd = amide; w = water), O—H_w_. .O_amd_ and O—H_w_⋯N_dma_ (dma = di­methyl­amino) hydrogen bonds (Table 1 ▸ and Fig. 2 ▸) link mol­ecules to form slabs lying parallel to (100). Within the slabs there are also C—H⋯O hydrogen bonds and C—H⋯π inter­actions present (Table 1 ▸). The N—H_amd_⋯O_w_, C—H_phen_⋯O_w_ (phen = phen­oxy), and the O—H_w_⋯ O_amd_, C—H_phen_⋯O_amd_ and C—H_phen_⋯O_w_ hydrogen bonds form 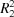
(7) and 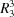
(7) ring motifs, respectively (Table 1 ▸ and Fig. 3 ▸). The slabs are linked *via* a pair of inversion-related C_phn_—H⋯N_phn_ (phn = phthalo­nitrile) hydrogen bonds, forming a three-dimensional structure (Table 1 ▸ and Fig. 4 ▸).

## Database survey   

A search of the Cambridge Structural Database (CSD, Version 5.36, last update May 2015; Groom & Allen, 2014 ▸) gave 29 hits for 4-phen­oxy­phthalo­nitrile, with no substituents in the positions *ortho* to the bridging O atom. The dihedral angle between the planes of the phthalo­nitrile and phen­oxy rings varies from *ca* 50.2–88.1°. In 4-phen­oxy­phthalo­nitrile itself (CSD refcode NIKFOD; Fang *et al.*, 2007 ▸) and two other similar compounds, namely 4-(*m*-tol­yloxy)phthalo­nitrile (JEVSAF; Ocak Ískeleli, 2007 ▸) and 4-(4-benzyl­oxyphen­oxy)phthalo­nitrile (IROSOX; Karadayı *et al.*, 2004 ▸), the dihedral angles between the two aromatic rings are *ca* 72.03, 68.18 and 71.31 °, respectively; similar to the same dihedral angle in the title compound, *viz.* 68.53 (16)°.

## Refinement   

The experimental details including the crystal data, data collection and refinement are summarized in Table 2 ▸. The water H atoms (H31 and H32) and the N—H H atom (H3) were located in a difference Fourier map and freely refined. The C-bound H atoms were positioned geometrically and constrained to ride on their parent atoms, with C—H = 0.93–0.97 Å and *U*_iso_(H) = 1.5*U*_eq_(C) for methyl H atoms and 1.2*U*_eq_(C) for the other H atoms.

## Synthesis and crystallization   

To a mixture of *N*,*N*-di­methyl­propane-1,3-di­amine (72 mg, 0.71 mmol) and K_2_CO_3_ (293 mg, 2.12 mmol) in dry tetra­hydro­furan (THF; 5 ml), stirred in an ice bath for 15 min, was added over a period of 40 min, 4-(3,4-di­cyano­phen­oxy)benzoyl chloride (200 mg, 0.71 mmol) in dry THF (5 ml). The reaction mixture was then stirred for 5 h at room temperature and monitored by thin-layer chromatography [THF–hexane (3:4 *v*/*v*) as a mobile phase on silica-gel plates]. The oily residue obtained was dissolved in MeOH. The solvent was evaporated slowly and colourless block-like crystals appeared in *ca* 10 d (yield 580 mg, 73%).

## Supplementary Material

Crystal structure: contains datablock(s) I, global. DOI: 10.1107/S2056989015014991/su5187sup1.cif

Structure factors: contains datablock(s) I. DOI: 10.1107/S2056989015014991/su5187Isup2.hkl

Supporting information file. DOI: 10.1107/S2056989015014991/su5187Isup3.cml

CCDC reference: 1418026

Additional supporting information:  crystallographic information; 3D view; checkCIF report

## Figures and Tables

**Table 1:** Hydrogen-bond geometry (, ) *Cg*2 is the centroid of the phenoxy ring C9C14.

*D*H*A*	*D*H	H*A*	*D* *A*	*D*H*A*
N3H3O3^i^	0.89(2)	1.99(2)	2.825(2)	155(2)
O3H31N4^ii^	0.97(3)	1.85(3)	2.808(2)	168(3)
O3H32O2^iii^	0.88(3)	1.93(3)	2.803(2)	176(3)
C13H13O3^i^	0.93	2.58	3.477(2)	162
C14H14O2^iv^	0.93	2.36	3.049(2)	131
C16H16*B* *Cg*2^v^	0.97	2.96	3.661(2)	130
C2H2N1^vi^	0.93	2.49	3.324(2)	149

Symmetry codes: (i) 
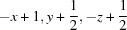
; (ii) 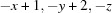; (iii) 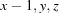; (iv) 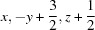; (v) 
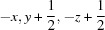
; (vi) 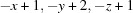.

**Table 2:** Experimental details

Crystal data	
Chemical formula	C_20_H_20_N_4_O_2_H_2_O
*M*_r_	366.42
Crystal system, space group	Monoclinic, *P*2_1_/*c*
Temperature (K)	100
*a*, *b*, *c* ()	12.9004(4), 10.5012(3), 14.1343(4)
()	99.819(5)
*V* (^3^)	1886.72(10)
*Z*	4
Radiation type	Mo *K*
(mm^1^)	0.09
Crystal size (mm)	0.41 0.21 0.12

Data collection	
Diffractometer	Bruker Kappa APEXII CCD area-detector diffractometer
Absorption correction	Multi-scan (*SADABS*; Bruker, 2012 ▸)
*T*_min_, *T*_max_	0.964, 0.989
No. of measured, independent and observed [*I* > 2(*I*)] reflections	11419, 4167, 3181
*R*_int_	0.048
(sin /)_max_ (^1^)	0.641

Refinement	
*R*[*F* ^2^ > 2(*F* ^2^)], *wR*(*F* ^2^), *S*	0.050, 0.134, 1.03
No. of reflections	4167
No. of parameters	258
H-atom treatment	H atoms treated by a mixture of independent and constrained refinement
_max_, _min_ (e ^3^)	0.30, 0.27

Computer programs: *APEX2* (Bruker, 2012 ▸), *SAINT* (Bruker, 2012 ▸), *SHELXS97* (Sheldrick, 2008 ▸), *SHELXL97* (Sheldrick, 2008 ▸), *ORTEP-3 for Windows* (Farrugia, 2012 ▸), *Mercury* (Macrae *et al.*, 2008 ▸), *WinGX* (Farrugia, 2012 ▸) and *PLATON* (Spek, 2009 ▸).

## supplementary crystallographic information

### Crystal data

**Table:**

C_20_H_20_N_4_O_2_·H_2_O	*F*(000) = 776
*M**_r_* = 366.42	*D*_x_ = 1.290 Mg m^−^^3^
Monoclinic, *P*2_1_/*c*	Mo *K*α radiation, λ = 0.71073 Å
Hall symbol: -P 2ybc	Cell parameters from 3102 reflections
*a* = 12.9004 (4) Å	θ = 2.4–27.6°
*b* = 10.5012 (3) Å	µ = 0.09 mm^−^^1^
*c* = 14.1343 (4) Å	*T* = 100 K
β = 99.819 (5)°	Block, colourless
*V* = 1886.72 (10) Å^3^	0.41 × 0.21 × 0.12 mm
*Z* = 4	

### Data collection

**Table:**

Bruker Kappa APEXII CCD area-detector diffractometer	4167 independent reflections
Radiation source: fine-focus sealed tube	3181 reflections with *I* > 2σ(*I*)
Graphite monochromator	*R*_int_ = 0.048
Detector resolution: 8.3333 pixels mm^-1^	θ_max_ = 27.1°, θ_min_ = 1.6°
φ and ω scans	*h* = −16→16
Absorption correction: multi-scan (*SADABS*; Bruker, 2012)	*k* = −13→8
*T*_min_ = 0.964, *T*_max_ = 0.989	*l* = −18→8
11419 measured reflections	

### Refinement

**Table:**

Refinement on *F*^2^	Primary atom site location: structure-invariant direct methods
Least-squares matrix: full	Secondary atom site location: difference Fourier map
*R*[*F*^2^ > 2σ(*F*^2^)] = 0.050	Hydrogen site location: inferred from neighbouring sites
*wR*(*F*^2^) = 0.134	H atoms treated by a mixture of independent and constrained refinement
*S* = 1.03	*w* = 1/[σ^2^(*F*_o_^2^) + (0.0573*P*)^2^ + 0.8262*P*] where *P* = (*F*_o_^2^ + 2*F*_c_^2^)/3
4167 reflections	(Δ/σ)_max_ < 0.001
258 parameters	Δρ_max_ = 0.30 e Å^−^^3^
0 restraints	Δρ_min_ = −0.27 e Å^−^^3^

### Special details

**Table:**

Geometry. All esds (except the esd in the dihedral angle between two l.s. planes) are estimated using the full covariance matrix. The cell esds are taken into account individually in the estimation of esds in distances, angles and torsion angles; correlations between esds in cell parameters are only used when they are defined by crystal symmetry. An approximate (isotropic) treatment of cell esds is used for estimating esds involving l.s. planes.
Refinement. Refinement of F^2^ against ALL reflections. The weighted R-factor wR and goodness of fit S are based on F^2^, conventional R-factors R are based on F, with F set to zero for negative F^2^. The threshold expression of F^2^ > 2sigma(F^2^) is used only for calculating R-factors(gt) etc. and is not relevant to the choice of reflections for refinement. R-factors based on F^2^ are statistically about twice as large as those based on F, and R- factors based on ALL data will be even larger.

### Fractional atomic coordinates and isotropic or equivalent isotropic displacement parameters (Å^2^)

**Table:**

	*x*	*y*	*z*	*U*_iso_*/*U*_eq_	
O1	0.67288 (10)	0.57054 (11)	0.44706 (9)	0.0266 (3)	
O2	0.89189 (10)	0.80540 (12)	0.12674 (9)	0.0268 (3)	
O3	0.03612 (13)	0.61391 (15)	0.10156 (11)	0.0390 (4)	
H31	0.069 (2)	0.647 (3)	0.050 (2)	0.067 (8)*	
H32	−0.008 (2)	0.676 (3)	0.107 (2)	0.069 (9)*	
N1	0.44298 (14)	0.98877 (15)	0.59877 (12)	0.0309 (4)	
N2	0.41361 (14)	0.74623 (16)	0.79739 (12)	0.0324 (4)	
N3	0.92472 (12)	0.97057 (14)	0.22694 (11)	0.0218 (3)	
H3	0.9178 (18)	1.006 (2)	0.2828 (17)	0.041 (6)*	
N4	0.84238 (12)	1.30422 (14)	0.03388 (11)	0.0256 (4)	
C1	0.62353 (14)	0.61497 (16)	0.51863 (12)	0.0209 (4)	
C2	0.57723 (14)	0.73408 (16)	0.51653 (12)	0.0207 (4)	
H2	0.5815	0.7908	0.4668	0.025*	
C3	0.52459 (13)	0.76621 (16)	0.59012 (12)	0.0202 (4)	
C4	0.51636 (13)	0.68108 (16)	0.66442 (12)	0.0204 (4)	
C5	0.56370 (15)	0.56311 (17)	0.66438 (13)	0.0244 (4)	
H5	0.5594	0.5056	0.7136	0.029*	
C6	0.61708 (14)	0.53065 (17)	0.59187 (13)	0.0242 (4)	
H6	0.6491	0.4512	0.5922	0.029*	
C7	0.47916 (15)	0.89059 (17)	0.59306 (13)	0.0233 (4)	
C8	0.45955 (14)	0.71679 (17)	0.73886 (13)	0.0234 (4)	
C9	0.71794 (13)	0.65280 (16)	0.38897 (12)	0.0207 (4)	
C10	0.70886 (13)	0.61852 (16)	0.29416 (12)	0.0212 (4)	
H10	0.6684	0.5486	0.2704	0.025*	
C11	0.76049 (13)	0.68930 (16)	0.23528 (12)	0.0208 (4)	
H11	0.7549	0.6665	0.1710	0.025*	
C12	0.82069 (13)	0.79391 (16)	0.26930 (12)	0.0188 (4)	
C13	0.82780 (14)	0.82712 (17)	0.36507 (12)	0.0217 (4)	
H13	0.8671	0.8979	0.3889	0.026*	
C14	0.77737 (14)	0.75652 (17)	0.42502 (12)	0.0232 (4)	
H14	0.7832	0.7784	0.4895	0.028*	
C15	0.88129 (13)	0.85836 (17)	0.20237 (12)	0.0196 (4)	
C16	0.99496 (14)	1.02932 (18)	0.16930 (13)	0.0253 (4)	
H16A	1.0372	0.9633	0.1465	0.030*	
H16B	1.0424	1.0863	0.2098	0.030*	
C17	0.93865 (15)	1.10365 (17)	0.08368 (13)	0.0246 (4)	
H17A	0.9893	1.1300	0.0441	0.029*	
H17B	0.8875	1.0487	0.0452	0.029*	
C18	0.88309 (14)	1.22012 (17)	0.11356 (13)	0.0246 (4)	
H18A	0.8252	1.1927	0.1444	0.030*	
H18B	0.9318	1.2676	0.1605	0.030*	
C19	0.80249 (17)	1.42049 (19)	0.07052 (16)	0.0351 (5)	
H19A	0.8586	1.4633	0.1118	0.053*	
H19B	0.7477	1.3998	0.1062	0.053*	
H19C	0.7748	1.4752	0.0178	0.053*	
C20	0.75924 (17)	1.2439 (2)	−0.03341 (16)	0.0389 (5)	
H20A	0.7852	1.1663	−0.0567	0.058*	
H20B	0.7366	1.3003	−0.0863	0.058*	
H20C	0.7009	1.2251	−0.0016	0.058*	

### Atomic displacement parameters (Å^2^)

**Table:**

	*U*^11^	*U*^22^	*U*^33^	*U*^12^	*U*^13^	*U*^23^
O1	0.0395 (8)	0.0162 (6)	0.0278 (7)	0.0019 (5)	0.0165 (6)	0.0011 (5)
O2	0.0318 (7)	0.0291 (7)	0.0212 (6)	−0.0018 (6)	0.0090 (5)	−0.0043 (5)
O3	0.0504 (10)	0.0340 (9)	0.0376 (9)	0.0149 (7)	0.0218 (7)	0.0141 (7)
N1	0.0405 (10)	0.0237 (9)	0.0304 (9)	0.0052 (7)	0.0115 (7)	0.0025 (7)
N2	0.0398 (10)	0.0268 (9)	0.0338 (9)	0.0026 (7)	0.0155 (8)	−0.0002 (7)
N3	0.0260 (8)	0.0203 (8)	0.0198 (8)	−0.0014 (6)	0.0058 (6)	0.0015 (6)
N4	0.0267 (8)	0.0210 (8)	0.0297 (8)	−0.0017 (6)	0.0062 (7)	0.0022 (6)
C1	0.0231 (9)	0.0180 (9)	0.0222 (9)	−0.0005 (7)	0.0052 (7)	−0.0022 (7)
C2	0.0246 (9)	0.0179 (9)	0.0197 (8)	−0.0005 (7)	0.0043 (7)	0.0033 (7)
C3	0.0206 (8)	0.0168 (8)	0.0225 (9)	−0.0002 (7)	0.0020 (7)	0.0002 (7)
C4	0.0209 (8)	0.0193 (9)	0.0217 (8)	−0.0024 (7)	0.0056 (7)	−0.0008 (7)
C5	0.0313 (10)	0.0191 (9)	0.0240 (9)	−0.0010 (8)	0.0083 (7)	0.0051 (7)
C6	0.0295 (10)	0.0164 (9)	0.0280 (9)	0.0031 (7)	0.0087 (8)	0.0031 (7)
C7	0.0281 (9)	0.0204 (9)	0.0221 (9)	−0.0020 (7)	0.0060 (7)	0.0011 (7)
C8	0.0291 (10)	0.0164 (9)	0.0256 (9)	−0.0009 (7)	0.0069 (8)	0.0025 (7)
C9	0.0230 (9)	0.0167 (8)	0.0232 (9)	0.0048 (7)	0.0067 (7)	0.0041 (7)
C10	0.0202 (9)	0.0178 (9)	0.0258 (9)	0.0007 (7)	0.0040 (7)	−0.0019 (7)
C11	0.0222 (9)	0.0216 (9)	0.0178 (8)	0.0022 (7)	0.0014 (7)	−0.0019 (7)
C12	0.0191 (8)	0.0178 (8)	0.0195 (8)	0.0056 (7)	0.0030 (6)	0.0016 (6)
C13	0.0260 (9)	0.0182 (9)	0.0210 (8)	−0.0005 (7)	0.0039 (7)	−0.0021 (7)
C14	0.0298 (10)	0.0229 (9)	0.0169 (8)	0.0007 (7)	0.0040 (7)	−0.0005 (7)
C15	0.0190 (8)	0.0206 (9)	0.0185 (8)	0.0040 (7)	0.0015 (6)	0.0016 (7)
C16	0.0213 (9)	0.0263 (10)	0.0289 (10)	−0.0025 (7)	0.0060 (7)	0.0037 (8)
C17	0.0261 (9)	0.0236 (10)	0.0251 (9)	−0.0018 (7)	0.0074 (7)	0.0022 (7)
C18	0.0240 (9)	0.0244 (10)	0.0259 (9)	−0.0022 (7)	0.0055 (7)	0.0009 (7)
C19	0.0359 (11)	0.0263 (10)	0.0452 (12)	0.0042 (9)	0.0128 (9)	0.0023 (9)
C20	0.0384 (12)	0.0285 (11)	0.0444 (13)	−0.0020 (9)	−0.0083 (10)	0.0059 (9)

### Geometric parameters (Å, º)

**Table:**

O1—C1	1.366 (2)	C11—C10	1.370 (2)
O1—C9	1.386 (2)	C11—H11	0.9300
O2—C15	1.233 (2)	C12—C11	1.383 (2)
O3—H31	0.96 (3)	C12—C13	1.386 (2)
O3—H32	0.87 (3)	C13—H13	0.9300
N1—C7	1.140 (2)	C14—C9	1.379 (2)
N3—C16	1.455 (2)	C14—C13	1.371 (2)
N3—H3	0.89 (2)	C14—H14	0.9300
N4—C18	1.457 (2)	C15—N3	1.325 (2)
N4—C19	1.454 (2)	C15—C12	1.489 (2)
N4—C20	1.452 (2)	C16—C17	1.517 (2)
C1—C2	1.384 (2)	C16—H16A	0.9700
C1—C6	1.376 (2)	C16—H16B	0.9700
C2—C3	1.378 (2)	C17—C18	1.514 (3)
C2—H2	0.9300	C17—H17A	0.9700
C4—C3	1.397 (2)	C17—H17B	0.9700
C4—C5	1.381 (2)	C18—H18A	0.9700
C5—C6	1.372 (3)	C18—H18B	0.9700
C5—H5	0.9300	C19—H19A	0.9600
C6—H6	0.9300	C19—H19B	0.9600
C7—C3	1.435 (2)	C19—H19C	0.9600
C8—N2	1.140 (2)	C20—H20A	0.9600
C8—C4	1.431 (3)	C20—H20B	0.9600
C10—C9	1.373 (2)	C20—H20C	0.9600
C10—H10	0.9300		
			
C1—O1—C9	121.43 (13)	C12—C13—H13	119.7
H31—O3—H32	99 (2)	C14—C13—C12	120.56 (16)
C15—N3—C16	120.40 (16)	C14—C13—H13	119.7
C15—N3—H3	120.2 (15)	C9—C14—H14	120.3
C16—N3—H3	119.0 (15)	C13—C14—C9	119.44 (16)
C19—N4—C18	109.66 (15)	C13—C14—H14	120.3
C20—N4—C18	111.73 (15)	O2—C15—N3	121.58 (16)
C20—N4—C19	109.46 (16)	O2—C15—C12	119.59 (16)
O1—C1—C2	123.13 (15)	N3—C15—C12	118.82 (15)
O1—C1—C6	115.67 (15)	N3—C16—C17	113.93 (15)
C6—C1—C2	121.10 (16)	N3—C16—H16A	108.8
C1—C2—H2	120.9	N3—C16—H16B	108.8
C3—C2—C1	118.13 (16)	C17—C16—H16A	108.8
C3—C2—H2	120.9	C17—C16—H16B	108.8
C2—C3—C4	121.43 (16)	H16A—C16—H16B	107.7
C2—C3—C7	120.07 (16)	C16—C17—H17A	109.2
C4—C3—C7	118.48 (16)	C16—C17—H17B	109.2
C5—C4—C3	118.90 (16)	C18—C17—C16	112.23 (15)
C5—C4—C8	121.15 (16)	C18—C17—H17A	109.2
C3—C4—C8	119.95 (16)	C18—C17—H17B	109.2
C4—C5—H5	119.9	H17A—C17—H17B	107.9
C6—C5—C4	120.11 (16)	N4—C18—C17	113.55 (15)
C6—C5—H5	119.9	N4—C18—H18A	108.9
C1—C6—H6	119.8	N4—C18—H18B	108.9
C5—C6—C1	120.32 (17)	C17—C18—H18A	108.9
C5—C6—H6	119.8	C17—C18—H18B	108.9
N1—C7—C3	177.56 (19)	H18A—C18—H18B	107.7
N2—C8—C4	179.2 (2)	N4—C19—H19A	109.5
C10—C9—O1	116.12 (16)	N4—C19—H19B	109.5
C10—C9—C14	121.15 (16)	N4—C19—H19C	109.5
C14—C9—O1	122.44 (15)	H19A—C19—H19B	109.5
C9—C10—H10	120.6	H19A—C19—H19C	109.5
C11—C10—C9	118.76 (16)	H19B—C19—H19C	109.5
C11—C10—H10	120.6	N4—C20—H20A	109.5
C10—C11—C12	121.46 (16)	N4—C20—H20B	109.5
C10—C11—H11	119.3	N4—C20—H20C	109.5
C12—C11—H11	119.3	H20A—C20—H20B	109.5
C11—C12—C13	118.63 (16)	H20A—C20—H20C	109.5
C11—C12—C15	117.69 (15)	H20B—C20—H20C	109.5
C13—C12—C15	123.50 (16)		
			
C9—O1—C1—C2	26.8 (2)	C4—C5—C6—C1	−0.2 (3)
C9—O1—C1—C6	−156.73 (16)	C11—C10—C9—O1	−173.73 (15)
C1—O1—C9—C10	−142.73 (16)	C11—C10—C9—C14	0.2 (3)
C1—O1—C9—C14	43.4 (2)	C12—C11—C10—C9	−0.2 (3)
C15—N3—C16—C17	84.1 (2)	C13—C12—C11—C10	−0.3 (3)
C20—N4—C18—C17	65.6 (2)	C15—C12—C11—C10	174.86 (15)
C19—N4—C18—C17	−172.83 (16)	C11—C12—C13—C14	0.9 (3)
O1—C1—C2—C3	176.48 (16)	C15—C12—C13—C14	−173.97 (16)
C6—C1—C2—C3	0.2 (3)	C13—C14—C9—O1	173.93 (16)
O1—C1—C6—C5	−176.17 (16)	C13—C14—C9—C10	0.4 (3)
C2—C1—C6—C5	0.4 (3)	C9—C14—C13—C12	−0.9 (3)
C1—C2—C3—C4	−0.9 (3)	O2—C15—N3—C16	−5.9 (2)
C1—C2—C3—C7	177.39 (16)	C12—C15—N3—C16	172.51 (14)
C5—C4—C3—C2	1.1 (3)	O2—C15—C12—C11	−13.1 (2)
C5—C4—C3—C7	−177.27 (16)	O2—C15—C12—C13	161.88 (16)
C8—C4—C3—C2	−178.93 (16)	N3—C15—C12—C11	168.50 (15)
C8—C4—C3—C7	2.7 (2)	N3—C15—C12—C13	−16.6 (2)
C3—C4—C5—C6	−0.5 (3)	N3—C16—C17—C18	66.6 (2)
C8—C4—C5—C6	179.52 (17)	C16—C17—C18—N4	170.87 (15)

### Hydrogen-bond geometry (Å, º)

Cg2 is the centroid of the phenoxy ring C9—C14.

**Table:**

*D*—H···*A*	*D*—H	H···*A*	*D*···*A*	*D*—H···*A*
N3—H3···O3^i^	0.89 (2)	1.99 (2)	2.825 (2)	155 (2)
O3—H31···N4^ii^	0.97 (3)	1.85 (3)	2.808 (2)	168 (3)
O3—H32···O2^iii^	0.88 (3)	1.93 (3)	2.803 (2)	176 (3)
C13—H13···O3^i^	0.93	2.58	3.477 (2)	162
C14—H14···O2^iv^	0.93	2.36	3.049 (2)	131
C16—H16*B*···*Cg*2^v^	0.97	2.96	3.661 (2)	130
C2—H2···N1^vi^	0.93	2.49	3.324 (2)	149

Symmetry codes: (i) −*x*+1, *y*+1/2, −*z*+1/2; (ii) −*x*+1, −*y*+2, −*z*; (iii) *x*−1, *y*, *z*; (iv) *x*, −*y*+3/2, *z*+1/2; (v) −*x*, *y*+1/2, −*z*+1/2; (vi) −*x*+1, −*y*+2, −*z*+1.
